# COVID-19 Stroke Apical Lung Examination Study 2: a national prospective CTA biomarker study of the lung apices, in patients presenting with suspected acute stroke (COVID SALES 2)

**DOI:** 10.1016/j.nicl.2024.103590

**Published:** 2024-03-15

**Authors:** T. Ratneswaren, N. Chan, J. Aeron-Thomas, S. Sait, O. Adesalu, M. Alhawamdeh, M. Benger, J. Garnham, L. Dixon, F. Tona, C. McNamara, E. Taylor, K. Lobotesis, E. Lim, O. Goldberg, N. Asmar, O. Evbuomwan, S. Banerjee, L. Holm-Mercer, J. Senor, Y. Tsitsiou, P. Tantrige, A. Taha, K. Ballal, A. Mattar, A. Daadipour, K. Elfergani, R. Barker, R. Chakravartty, A.G. Murchison, B.J. Kemp, R. Simister, I. Davagnanam, O.Y. Wong, D. Werring, A. Banaras, M. Anjari, J.C.L. Rodrigues, C.A.S. Thompson, I.R. Haines, T.A. Burnett, R.E.Y. Zaher, V.L. Reay, M. Banerjee, C.S.L. Sew Hee, A.P. Oo, A. Lo, P. Rogers, T. Hughes, A. Marin, S. Mukherjee, H. Jaber, E. Sanders, S. Owen, M. Bhandari, S. Sundayi, A. Bhagat, M. Elsakka, O.H. Hashmi, M. Lymbouris, Y. Gurung-Koney, M. Arshad, I. Hasan, N. Singh, V. Patel, M. Rahiminejad, T.C. Booth, on behalf of the RADIANT Group

**Affiliations:** a Charing Cross Hospital, London, UK; b Addenbrooke’s Hospital, Cambridge, UK; c Royal London Hospital, London, UK; d King’s College Hospital, London, UK; e Princess Royal University Hospital, Orpington, UK; f Frimley Park Hospital, Surrey, UK; g John Radcliffe Hospital, Oxford, UK; h University College Hospital, London, UK; i Comprehensive Stroke Service, National Hospital for Neurology and Neurosurgery, University College Hospitals NHS Foundation Trust, London, UK; j Stroke Research Centre, UCL Queen Square Institute of Neurology, London, UK; k Lysholm Department of Neuroradiology, National Hospital for Neurology and Neurosurgery, University College London Hospitals NHS Foundation Trust, UK; l Royal United Hospital, Bath, UK; m Southampton General Hospital, Southampton, UK; n Cardiff and Vale University Health Board, Cardiff, UK; o Watford General Hospital, Watford, UK; p Norfolk and Norwich University Hospital, Norwich, UK; q St Thomas’ Hospital, London, UK; r School of Biomedical Engineering & Imaging Sciences, King’s College London, London, UK

**Keywords:** Stroke, Lung apices, COVID-19, Diagnostic biomarker

## Abstract

**Background:**

Apical ground-glass opacification (GGO) identified on CT angiography (CTA) performed for suspected acute stroke was developed in 2020 as a coronavirus-disease-2019 (COVID-19) diagnostic and prognostic biomarker in a retrospective study during the first wave of COVID-19.

**Objective:**

To prospectively validate whether GGO on CTA performed for suspected acute stroke is a reliable COVID-19 diagnostic and prognostic biomarker and whether it is reliable for COVID-19 vaccinated patients.

**Methods:**

In this prospective, pragmatic, national, multi-center validation study performed at 13 sites, we captured study data consecutively in patients undergoing CTA for suspected acute stroke from January-March 2021. Demographic and clinical features associated with stroke and COVID-19 were incorporated. The primary outcome was the likelihood of reverse-transcriptase-polymerase-chain-reaction swab-test-confirmed COVID-19 using the GGO biomarker. Secondary outcomes investigated were functional status at discharge and survival analyses at 30 and 90 days. Univariate and multivariable statistical analyses were employed.

**Results:**

CTAs from 1,111 patients were analyzed, with apical GGO identified in 8.5 % during a period of high COVID-19 prevalence. GGO showed good inter-rater reliability (Fleiss κ = 0.77); and high COVID-19 specificity (93.7 %, 91.8–95.2) and negative predictive value (NPV; 97.8 %, 96.5–98.6). In subgroup analysis of vaccinated patients, GGO remained a good diagnostic biomarker (specificity 93.1 %, 89.8–95.5; NPV 99.7 %, 98.3–100.0). Patients with COVID-19 were more likely to have higher stroke score (NIHSS (mean +/- SD) 6.9 +/- 6.9, COVID-19 negative, 9.7 +/- 9.0, COVID-19 positive; p = 0.01), carotid occlusions (6.2 % negative, 14.9 % positive; p = 0.02), and larger infarcts on presentation CT (ASPECTS 9.4 +/- 1.5, COVID-19 negative, 8.6 +/- 2.4, COVID-19 positive; p = 0.00). After multivariable logistic regression, GGO (odds ratio 15.7, 6.2–40.1), myalgia (8.9, 2.1–38.2) and higher core body temperature (1.9, 1.1–3.2) were independent COVID-19 predictors. GGO was associated with worse functional outcome on discharge and worse survival after univariate analysis. However, after adjustment for factors including stroke severity, GGO was not independently predictive of functional outcome or mortality.

**Conclusion:**

Apical GGO on CTA performed for patients with suspected acute stroke is a reliable diagnostic biomarker for COVID-19, which in combination with clinical features may be useful in COVID-19 triage.

## Highlights

• GGO in the lung apices on head and neck CTA for stroke is a reliable biomarker for COVID-19, with high specificity.
• GGO is not an independent predictor of outcome in suspected stroke patients.
• Apical GGO on CTA may be useful in combination with clinical features, in COVID-19 triage.

## 1 Introduction

The worldwide coronavirus disease 2019 (COVID-19) pandemic has resulted in patients with COVID-19 attending emergency departments (ED) and being admitted to hospital wards, either due to COVID-19 or for co-existing medical conditions. Scientists warn that circulation of Severe Acute Respiratory Syndrome coronavirus 2 (SARS-CoV-2), perhaps with seasonal epidemic peaks, is likely to have a continued important disease burden (Telenti et al., 2021). The most recent peak started in December 2022 in China, compounded by incomplete vaccination coverage (Leung et al., 2023). There remains a global need for COVID-19 triage to identify patients presenting to hospitals with COVID-19, to limit the spread of infection. Reverse transcriptase polymerase chain reaction swab tests (RT-PCR) for SARS-CoV-2 are used in emergency departments to aid decision making for COVID-19 triage, however these often take several hours to return and sometimes are inconclusive (Yang et al., 2021).

Patients with suspected acute stroke caused by large vessel occlusion are potentially eligible for mechanical thrombectomy, and CTA from the aortic arch to cranial vertex is performed routinely in the imaging work up. Early in the pandemic, ground-glass opacification (GGO) in the lung apices on CTA, performed for patients with suspected acute stroke, was identified as a COVID-19 diagnostic biomarker with good sensitivity (75 %) and specificity (81 %) that was available before RT-PCR results (Siddiqui et al., 2020). Furthermore, it was demonstrated that apical GGO seen on CTA could be used as a prognostic biomarker because the presence of GGO was an independent predictor of 30-day mortality (Siddiqui et al., 2020).

A review of the lung apices on CTA is therefore of potential value to facilitate the COVID-19 triage of patients presenting with suspected acute stroke undergoing CTA in their routine clinical work up and is being used by many centers globally to assist immediate patient management. However, the study that developed the biomarker used retrospective data limited to three hyperacute stroke units in London during the first UK wave of COVID-19. During this time, the wild-type variant predominated (Whitaker et al., 2022). With the emergence of new strains of COVID-19 and the introduction of population-wide vaccination, we hypothesized that apical GGO seen on CTA remains a reliable COVID-19 diagnostic and prognostic biomarker. The primary objective of this study was to prospectively validate after a year of the pandemic, whether apical GGO seen on CTA in patients presenting with suspected acute stroke, is a reliable COVID-19 diagnostic and prognostic biomarker. A secondary objective was to identify in this cohort of patients, other co-variates (routinely collected on admission to hospital) which can serve as potential biomarkers for COVID-19 diagnosis and prognosis.

## 2 Methods

### 2.1 Study design

We performed a prospective, pragmatic, UK-wide, multicenter study which was designed and reported according to STARD 2015 guidelines for reporting of diagnostic accuracy studies (Bossuyt et al., 2015). The UK’s National Health Research Authority and Research Ethics Committee approved the study and requested that patient consent was not obtained given the COVID emergency (IRAS 284437). The study was portfolio-adopted by the UK’s National Institute of Health Care and Research (NIHR). Thirteen sites were enrolled: Cambridge University Hospitals NHS Foundation Trust, Cardiff and Vale University Health Board, Frimley Health NHS Foundation Trust, Guy’s and St Thomas’ NHS Foundation Trust, Imperial College NHS Foundation Trust, King’s College Hospital NHS Foundation Trust, Norfolk and Norwich University Hospital NHS Foundation Trust, Oxford University NHS Foundation Trust, Princess Royal University Hospital, Royal United Hospitals Bath NHS Foundation Trust, University College London Hospital, University Hospital Southampton NHS Foundation Trust and West Hertfordshire NHS Foundation Trust.

### 2.2 Participants

Consecutive patients attending the emergency department (ED) at the recruitment sites from 1st January to 31st March 2021 with suspected acute stroke and who underwent CTA at presentation were recruited. This was a period of high disease incidence (5.38 per 10,000 per day) (Office for National Statistics, 2022). The index test under investigation was the CTA. RT-PCR results, of COVID-19 RT-PCR swabs performed within 12 hours of admission, were used as the reference standard to compare CTA results against, as this test has the highest sensitivity (85–98 %) and specificity (99.9 %) of all clinically available tests (Office for National Statistics, 2022).

Adult patients with a) suspected acute ischemic stroke, and b) undergoing CTA from aortic arch to cranial vertex, were included in the study. Exclusion criteria were a) inpatients with suspected stroke arising after admission, b) patients with CTA performed more than four hours after their first non-contrast CT head (NCCT) performed in ED, and c) patients with known COVID-19 infection prior to ED admission.

### 2.3 Data collection and test methods

**Fig. 1:**
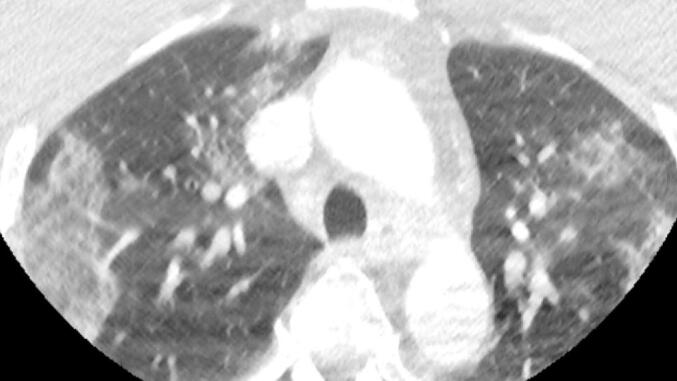
GGO in the lung apices of a patient with COVID-19.

Prior to the study, sites received training materials regarding the assessment of apical GGO for CTA and the Alberta Stroke Program Early CT score (ASPECTS) for NCCT (Siddiqui et al., 2020). Fig. 1 demonstrates representative GGO in the lung apices.

NCCT and CTA, were read by two radiologists independently (at least one of whom had completed the UK’s radiology licensure examination (Fellowship of the Royal College of Radiologists); similar to board certification in US). Readers were blinded to clinical or RT-PCR data. If there were discrepant imaging readings, consensus was then reached between the two readers, or a third reader undertook an independent review.

Clinical and demographic data were extracted from paper and electronic patient records. Demographic data collected included age, sex and self-reported ethnicity. Baseline clinical data collected included risk factors for stroke, clinical features for stroke and COVID-19, and COVID-19 vaccination status. Treatment data was collected for thrombolysis and thrombectomy. Outcome data, including discharge modified Rankin Scale (mRS) and in-patient mortality data, were also collected.

### 2.4 Data analysis

Descriptive statistics were generated to summarize the data. Comparative statistics were used to compare cases (RT-PCR positive or GGO positive) and controls (RT-PCR negative or GGO negative, respectively). For univariate analyses, the chi-squared test (χ^2^) was used for categorical variables and Student’s *t*-test was used for parametric continuous data. Multivariable logistic regression was performed to predict the likelihood of a positive RT-PCR result.

For imaging analysis, the level of agreement between independent readers was measured using the Fleiss-κ coefficient and interpreted according to standard interpretation guidelines (Landis and Koch, 1977).

Multivariable logistic regression and ordinal logistic regression (shift analysis) were used to identify whether functional outcome, measured by discharge mRS, were independent of RT-PCR result or presence of GGO.

Survival analyses were performed at 30 and 90 days after the onset of stroke symptoms. Kaplan-Meier survival curves were generated to demonstrate survival of cases and controls. Univariate analysis was performed using the log-rank test of equality for categorical variables and the univariate cox proportional hazard regression for continuous variables. Statistically significant predictors were selected to build the multivariable cox regression model.

P < 0.05 was considered statistically significant. Statistical analyses were performed using STATA Version 17 (StataCorp, College Station, US).

## 3 Results

### 3.1 Participants

**Fig. 2:**
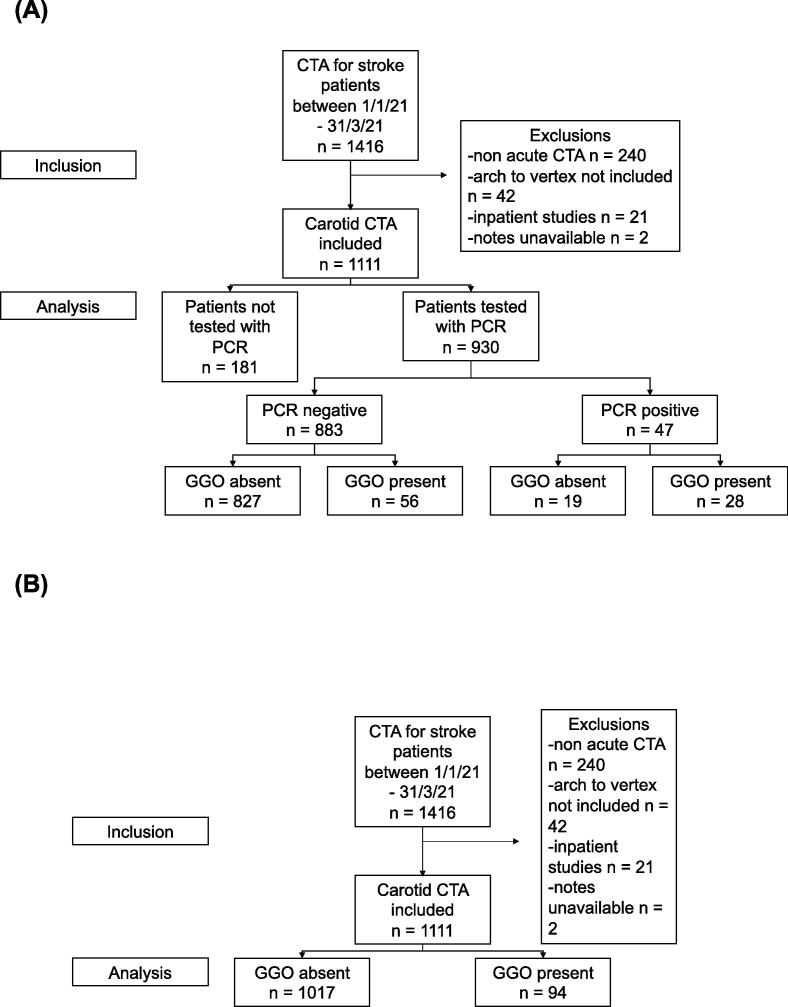
**(a)** Flow diagram demonstrating patient selection for inclusion of patients with CTA in acute suspected stroke stratified by RT-PCR result and the absence or presence of GGO. **(b)** Flow diagram demonstrating patient selection for inclusion of patients with CTA in acute suspected stroke, stratified by the absence or presence of GGO alone. CTA = CT Angiography. GGO = Ground-Glass Opacification. RT-PCR = Reverse Transcriptase Polymerase Chain Reaction swab test.

We identified 1,111 patients for inclusion in the study, of which 930 underwent RT-PCR testing (Fig. 2). We analyzed two groups consisting of firstly, patients who underwent CTA (n = 1111) and secondly, a large subgroup of patients who additionally underwent RT-PCR (n = 930). The grouping followed previous biomarker development methodology (Siddiqui et al., 2020).

### 3.2 Patient characteristics

Imaging data was acquired for 1,111 patients, of which 8.5 % demonstrated apical GGO. An RT-PCR result was recorded in 83.7 % of patients, of which 5.1 % tested positive for COVID-19. Demographic details for patients are included in Table 1 stratified by RT-PCR result. There was no statistically significant difference in age, sex, ethnicity or baseline functional status between patients by RT-PCR result. In terms of imaging findings, apical GGO, carotid occlusion, infarct on presentation CT and lower Alberta Stroke Program Early CT score (ASPECTS) scores were associated with a positive RT-PCR. With regards to clinical features, patients with COVID-19 were more likely to present with lower Glasgow Coma Scale (GCS), higher National Institute of Health Stroke Score (NIHSS), symptomatic subjective perception of fever, a higher presentation core body temperature, cough, fatigue, shortness of breath, lower oxygen saturations and myalgia compared with patients with a negative RT-PCR result.

**Table 1:** Patient characteristics stratified by RT-PCR result.

	All Patients (n = 1111)	RT-PCR negative (n = 883)	RT-PCR positive (n = 47)	*P* value
**Demographics**				
Age (mean, years)	67.9 ± 16.2 (1111/1111)	68.9 ± 15.5 (883/883)	66.8 ± 17.7 (47/47)	0.38
Sex (female)	48.2 (535/1111)	49.0 (883/883)	46.8 (47/47)	0.77
Ethnicity				
White	68.9 (710/1031)	69.4 (571/823)	68.9 (31/45)	0.95
Mix	1.4 (14/1031)	1.3 (11/823)	0.0 (0/45)	0.44
Asian	4.9 (51/1031)	5.0 (41/823)	8.9 (4/45)	0.22
Black	6.6 (68/1031)	7.0 (57/823)	4.4 (2/45)	0.56
Unknown	15.9 (164/1031)	14.6 (120/823)	15.6 (7/45)	0.57

**Baseline functional status**				
modified Rankin Scale (mRS) (mean)	0.7 ± 1.2 (1082/1111)	0.7 ± 1.2 (860/883)	0.9 ± 1.4 (46/47)	0.45
**Imaging findings on CT and CTA**				
GGO	8.5 (94/1111)	6.3 (56/883)	59.6 (28/47)	*0.00*
Carotid occlusion	6.7 (74/1111)	6.2 (55/883)	14.89 (7/47)	*0.02*
Large vessel occlusion	13.0 (144/1111)	12.8 (113/883)	19.1 (9/47)	0.21
Medium vessel occlusion	9.8 (109/1111)	10.4 (92/883)	10.6 (5/47)	0.96
Tandem occlusion	2.8 (30/1111)	2.49 (22/883)	4.3 (2/47)	0.46
Clot length (mm)	27.4 ± 65.8 (211/1111)	28.02 ± 70.4 (185/883)	23.0 ± 39.4 (14/47)	0.79
Acute infarct	23.4 (260/1111)	23.8 (210/883)	40.4 (19/47)	*0.01*
ASPECTS score (mean)	9.4 ± 1.5 (1111/1111)	9.4 ± 1.5 (883/883)	8.6 ± 2.4 (47/47)	*0.00*

**Symptoms**				
Subjective fever	3.4 (34/1008)	2.3 (18/800)	33.3 (14/42)	*0.00*
Cough	5.4 (52/984)	4.1 (32/782)	34.2 (14/40)	*0.00*
Fatigue	6.9 (61/887)	5.96 (42/705)	29.3 (12/41)	*0.00*
Shortness of breath	4.4 (40/915)	3.0 (22/729)	26.8 (11/41)	*0.00*
Myalgia	4.5 (40/884)	3.9 (27/702)	25.0 (10/40)	*0.00*

**Signs**				
GCS (mean)	14.2 ± 1.9 (1037/1111)	14.2 ± 1.9 (831/883)	13.3 ± 2.9 (47/47)	*0.00*
NIHSS (mean)	6.6 ± 7.0 (989/1111)	6.9 ± 6.9 (791/883)	9.7 ± 9.0 (42/47)	*0.01*
Heart rate (beats per minute)	81.8 ± 16.8 (1059/1111)	81.6 ± 16.6 (853/883)	85.6 ± 15.3 (47/47)	0.10
Respiratory rate (breaths per minute)	18.4 ± 4.0 (1056/1111)	18.45 ± 4.2 (853/883)	18.78 ± 3.0 (46/47)	0.60
Systolic blood pressure (mmHg)	150.8 ± 28.5 (1063/1111)	151.39 ± 28.0 (855/883)	146.6 ± 27.4 (47/47)	0.26
Diastolic blood pressure (mmHg)	84.8 ± 17.8 (1061/1111)	84.8 ± 17.4 (853/883)	81.5 ± 15.6 (47/47)	0.21
Core body temperature (mean, ℃)	36.7 ± 1.2 (1054/1111)	36.6 ± 1.3 (850/883)	37.2 ± 1.1 (47/47)	*0.00*
Oxygen saturations (mean, %)	97.1 ± 2.2 (1059/1111)	97.2 ± 2.1 (853/883)	95.3 ± 3.0 (47/47)	*0.00*
Supplementary oxygen required (%)	4.3 (48/1111)	4.2 (37/883)	6.4 (3/47)	0.44

**Comorbidities**				
Hypertension	51.3 (564/1111)	52.1 (456/875)	48.9 (23/47)	0.67
Diabetes	21.9 (241/1099)	22.6 (198/875)	27.7 (13/47)	0.42
Cardiovascular disease	21.6 (237/1099)	21.3 (186/875)	21.3 (10/47)	1.00
Atrial fibrillation	17.1 (188/1098)	18.3 (160/874)	8.5 (4/47)	0.09
Hypercholesterolemia	25.9 (282/1087)	27.9 (241/865)	19.2 (9/47)	0.19
Sickle cell disease	0.8 (8/1071)	0.94 (8/850)	0.0 (0/47)	0.50
Body Mass Index	27.3 ± 9.9 (592/1111)	27.7 ± 10.6 (485/883)	29.0 ± 7.3 (29/47)	0.16
Smoking status	32.2 (326/1013)	33.1 (8/850)	26.7 (12/45)	0.37
**Past medical history of stroke**	27.3 (286/1082)	29.0 (241/861)	17.0 (8/47)	0.10
**Family history of stroke**	5.4 (54/995)	5.4 (42/782)	2.2 (1/46)	0.34
**At least one vaccine dose**	58.5 (419/716)	59.2 (346/584)	28.0 (7/25)	*0.00*
**Treatment**				
Thrombolysis	18.6 (207/1111)	20.4 (180/883)	17.0 (8/47)	0.58
Time from door to thrombolysis (minutes, mean)	64.6 ± 49.6 (190/1111)	63.1 ± 50.2 (164/883)	88 ± 23.6 (7/47)	0.19
Thrombectomy	5.6 (62/1111)	5.7 (50/883)	2.1 (1/47)	0.30

Data are mean ± standard deviation or % (n/n).

Data in *italics* are statistically significant at the p < 0.05 significance level.

RT-PCR = Reverse Transcriptase Polymerase Chain Reaction swab test. mRS = modified Rankin Scale. CT = Computed Tomography. CTA = CT Angiography. GGO = Ground-Glass Opacification. ASPECTS = Alberta Stroke Program Early CT score. GCS = Glasgow Coma Scale. NIHSS = National Institute of Health Stroke Score.

Carotid occlusion is defined as occlusion of the common carotid artery or the cervical, petrous or cavernous segments of the internal carotid artery.

Large vessel occlusion is defined as intra-cranial occlusion of a major intracranial vessel including the distal internal carotid artery (ophthalmic, posterior communicating, anterior choroidal and terminal segments), the middle cerebral artery at M1 (prior to the bifurcation), anterior cerebral artery at A1, vertebral artery at V4, basilar artery or posterior cerebral artery at P1.

Medium–vessel occlusion is defined as vascular occlusion of the middle cerebral artery (M2/3), anterior cerebral artery (A2/3) or posterior cerebral artery (P2/3).

For patients stratified by apical GGO absence or presence, similar group comparisons were demonstrated regarding demographics, imaging findings and clinical features (Table S1).

There was a higher proportion of unvaccinated patients amongst the patients with a positive RT-PCR result compared with those with a negative RT-PCR, but no difference for GGO absence or presence.

There was no significant difference in rate of thrombolysis according to RT-PCR status or GGO presence (Tables 1 and S1). Furthermore, there was no significant difference in time from door to thrombolysis between patients with a positive RT-PCR result or in the presence of apical GGO. Similarly, the rate of thrombectomy was independent of RT-PCR status or GGO presence. There was little completed documentation of thrombectomy time data and therefore this was excluded.

### 3.3 Analysis of CTA as a diagnostic biomarker

Apical GGO had a very high negative predictive value (NPV) 97.8 % (96.5––98.6 (95 % CI)), which in subgroup analysis was 99.7 % (98.3–100) and 97.4 % (94.4–99.0) for vaccinated and unvaccinated patients respectively (Table 2). Further subgroup analysis showed a high NPV of apical GGO regardless of seniority (assessment by junior and senior radiologists gave NPVs of 97.1 % (95.5–98.3) and 97.8 % (96.8–98.6), respectively). The inter-rater agreement between readers for the presence of apical GGO was substantial (96.2 %; Fleiss κ = 0.77 ± 0.030 (±SD), p = 0.00) (Landis and Koch, 1977).

**Table 2:** Analysis of ground-glass opacification as a diagnostic biomarker in patients with a RT-PCR result.

	PPV % (95 % CI)	NPV % (95 % CI)	Sensitivity % (95 % CI)	Specificity % (95 % CI)
**Population as a whole (930/1111)**	33.3 (23.4–44.5)	97.8 (96.5–98.6)	59.6 (44.3–73.6)	93.7 (91.8–95.2)
**Unvaccinated subgroup** **(256/609)**	46.2 (26.6 – 66.6)	97.4 (94.4–99.0)	66.7 (41.0 – 86.7)	94.1 (90.3 – 96.7)
**Vaccinated subgroup** **(353/609)**	20.0 (7.7–38.6)	99.7 (98.3–100)	85.7 (42.1–99.6)	93.1 (89.8–95.5)
**Junior Radiologist (687/1860)**	30.3 (19.6–42.9)	97.1 (95.5–98.3)	52.6 (35.8–69.0)	92.9 (90.7–94.8)
**Senior Radiologist (1173/1860)**	30.8 (22.3–40.5)	97.8 (96.8–98.6)	58.9 (45.0–71.9)	93.4 (92.8–94.8)

Data are % with 95% confidence interval.

PPV = positive predictive value. NPV = negative predictive value.

Junior radiologists are radiology trainees (UK registrar grade; US resident equivalent) who have not obtained the UK’s radiology licensure examination (Fellowship of the Royal College of Radiologists which is similar to board certification in the US). Senior radiologists are those who have passed the UK’s radiology licensure examination.

### 3.4 Prediction of positive COVID-19 status

Multivariate logistic regression was performed on all demographic, clinical and imaging co-variates which showed group comparison differences of p < 0.05 on univariate analysis for the prediction of positive RT-PCR. Adjustment was therefore made for carotid occlusion, infarct and ASPECTS score on presentation CT, NIHSS, GCS, symptomatic subjective perception of fever, cough, fatigue, shortness of breath, myalgia, core body temperature, oxygen saturations and GGO. After adjustment for variables, myalgia, presentation core body temperature and GGO were independent predictors of a positive RT-PCR result (Table 3). Patients with GGO on CTA had an odds ratio (OR) of 15.7 (6.2–40.1) for a positive RT-PCR result when compared with patients without GGO.

**Table 3:** Adjusted odds ratio for obtaining a positive RT-PCR result.

	OR (95 % CI)	*P* value
**Carotid occlusion**	1 (0.2–5.3)	1.00
**Acute infarct**	2.3 (0.6–8.2)	0.20
**ASPECTS**	1.3 (0.9–1.9)	0.19
**NIHSS**	1.0 (1.0–1.1)	0.40
**GCS**	1.0 (0.7–1.4)	0.88
**Subjective fever**	1.7 (0.4–7.3)	0.49
**Cough**	2.4 (0.7–8.9)	0.19
**Fatigue**	0.5 (0.1–2.6)	0.38
**Shortness of breath**	1.9 (0.3–12.6)	0.49
**Myalgia**	8.9 (2.1–38.2)	*0.00*
**Core body temperature**	1.9 (1.1–3.2)	*0.01*
**Oxygen saturations**	0.9 (0.7–1.1)	0.13
**GGO**	15.7 (6.2–40.1)	*0.00*

Data are presented as odds ratios with 95% confidence intervals.

Data in *italics* are statistically significant at the p < 0.05 significance level.

ASPECTS = Alberta Stroke Program Early CT score. NIHSS = National Institute of Health Stroke Score. GCS = Glasgow Coma Scale. GGO = Ground-Glass Opacification.

### 3.5 Functional outcome

On univariate analysis, a worse functional outcome at discharge (independent (mRS = 0–2) compared to dead/dependent (mRS > 2)) was seen in patients with a positive RT-PCR (OR 0.54, 0.29–0.99 (95 % CI), p = 0.046), and apical GGO (OR 0.48, 0.31–0.74, p = 0.001). However, RT-PCR and GGO were not independent predictors of death/dependency after multivariate adjustment for those factors which had shown group comparison differences of p < 0.05 on univariate analysis for independent versus dependent/dead outcome at discharge (Table S2).

**Fig. 3:**
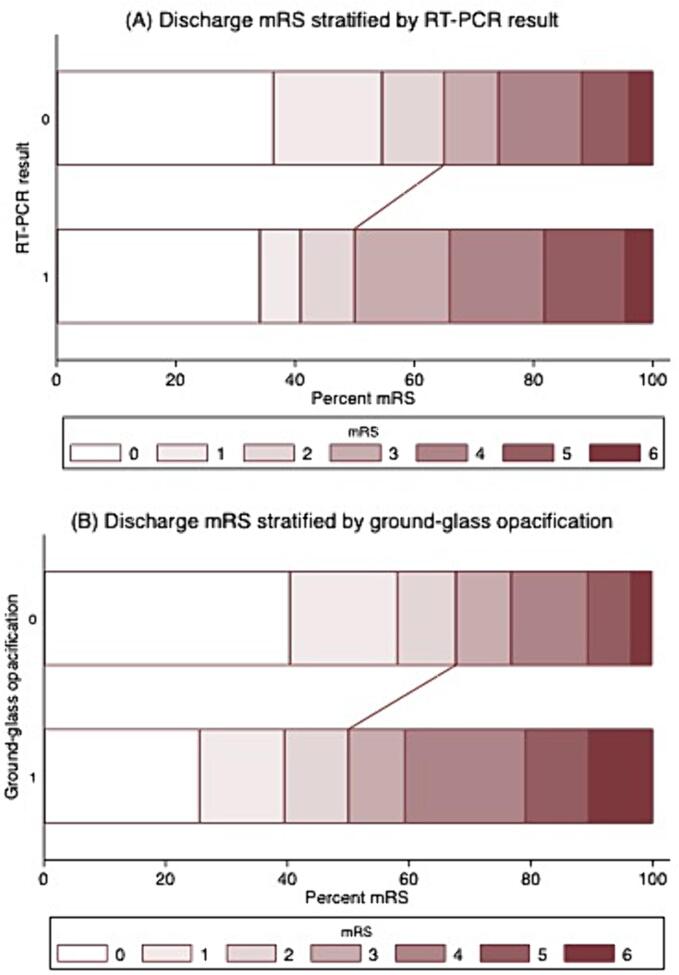
Grotta plots demonstrating functional outcomes at discharge stratified (a) by RT-PCR swab result and (b) in the absence or presence of GGO. The plots demonstrate a shift to dependency on discharge (mRS > 2) in patients with a positive RT-PCR result and in the presence of GGO. GGO = ground-glass opacification. RT-PCR = reverse transcriptase polymerase chain reaction swab test. mRS = modified Rankin Scale.

When all mRS levels were considered in an ordinal shift analysis (Fig. 3), a worse functional outcome at discharge was seen in patients with apical GGO on univariate analysis (OR 1.79, 1.10–2.68 (95 % CI), p = 0.005). After multivariate adjustment for those factors which had shown group comparison differences of p < 0.05 on univariate analysis for mRS shift at discharge (Table S2), RT-PCR was an independent predictor of functional outcome, however GGO was not an independent predictor.

There was no statistically significant difference in rates of symptomatic hemorrhage or length of stay post-admission in patients who tested positive on RT-PCR or in the presence of GGO on CTA (Table S2).

### 3.6 Survival analysis

**Fig. 4:**
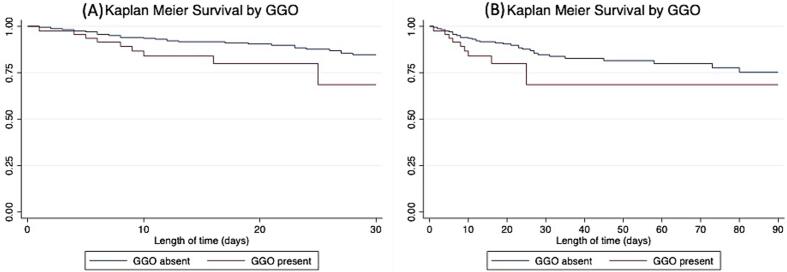
Kaplan Meier survival curves demonstrating survival for patients in the absence and presence of GGO at 30 days (a) and 90 days (b).

Univariate survival analysis was performed at 30 and 90 days (Tables S3 and S4). Those demographic, clinical and imaging co-variates, which had shown group comparison survival differences (p < 0.05) were used to build multivariable cox regression models for 30 and 90 day outcomes (Tables S5 and S6). Variables associated with survival differences at 30 days in univariate analysis were age, imaging findings (GGO (Fig. 4), carotid occlusion, large vessel occlusion, tandem occlusion, infarct, ASPECTS score) and clinical features (GCS, NIHSS, cough, shortness of breath, respiratory rate, requirement for supplementary oxygen and oxygen saturations). Similar variables were associated with survival differences at 90 days.

Variables which demonstrate statistically significant independent association with 30- and 90-day survival after multivariable regression were supplementary oxygen (30 day OR = 5.07, 2.03–12.65 (95 % CI), p = 0.001; 90 days OR = 4.33, 1.81–10.39, p = 0.001) and oxygen saturations (30 day OR = 0.90.83–0.98, p = 0.013; 90 day OR = 0.92, 0.85–0.99, p = 0.022).

All multivariate analyses were performed using listwise deletion. Complete-case analyses was judged to be a proportionate strategy with minimal bias (Hughes et al., 2019).

## 4 Discussion

### 4.1 Summary of findings

In this prospective, pragmatic, multicenter, national study, we have demonstrated that apical GGO in those patients undergoing CTA performed for suspected acute stroke has a high specificity (93.7 %) and NPV (97.8 %) as a COVID-19 diagnostic biomarker during a period of high prevalence of COVID-19. In contrast, all other contemporaneous demographic, clinical, and imaging features available at the time of CTA were not helpful in the early identification of COVID-19 other than myalgia and higher core body temperature.

We have demonstrated good inter-rater reliability between readers suggestive that apical GGO is a reliable biomarker. Furthermore, we have demonstrated that the biomarker is relatively easy to interpret, with similar NPV between junior and senior radiologists.

### 4.2 Comparison with studies worldwide

Apical GGO on CTA performed for suspected acute stroke was developed as a COVID-19 diagnostic and prognostic biomarker in a retrospective study performed at three London hospitals during the first wave of COVID-19 in a non-vaccinated population (Siddiqui et al., 2020). Our prospective validation study has confirmed that the apical GGO biomarker is a reliable biomarker when applied to a large number of institutions (13 institutions) located throughout the UK, when performed at a later time point in the pandemic, and when the UK population was predominantly vaccinated and the virus had mutated. Compared with the biomarker development study, we have demonstrated (a) a specificity of 94 % (92–95, 95 % CI) compared with 81 %, (71–88) previously and (b) a negative predictive value 98 % (97–99) compared with 90 % (79–95 95 % CI) previously. Furthermore, we have investigated a large range of potential COVID-19 predictors compared with other studies.

### 4.3 Study explanations and relevance from a national and international perspective

The findings are of clinical use for the COVID-19 triage of patients from the emergency department, where the absence of GGO can be helpful in indicating that a patient is highly likely to be negative for COVID-19, especially if vaccinated. Whilst GGO has the highest likelihood for COVID-19 diagnosis of any biomarker, we have also identified that myalgia and higher core body temperature on admission were associated with a higher rate of a positive RT-PCR result for patients with suspected stroke and these could also be assessed during COVID-19 triage. However, an accurate threshold for core body temperature has not been determined.

In hospital networks where there is a “hub and spoke” arrangement for stroke patient transfer, the presence or absence of GGO on CTA can be determined during tele-radiology thrombectomy assessment, allowing EDs and stroke departments to plan the patient pathway appropriately, pending the RT-PCR result.

If the patient is vaccinated and the lung apices do not contain GGO, based on our results, many hospitals may wish to process the patient as COVID-19 negative. If GGO is seen, whilst the positive predictive value for the test is low (33 %), more resources could be directed towards processing a RT-PCR swab as quickly as possible, whilst all personnel in close proximity to the patient should use appropriate personal protective equipment, and patients should be isolated after acute treatment pending the RT-PCR result.

Our finding that patients with COVID-19 have worse stroke scores (NIHSS 9.7 ± 9.0) compared with patients without COVID-19 (NIHSS 6.9 ± 6.9) is consistent with previous findings that COVID-19 infection is associated with more severe stroke at presentation (Perry et al., 2021). Moreover, it is important to note that patients with apical GGO are also associated with higher stroke scores (NIHSS 9.0 ± 8.6) compared with patients without GGO (NIHSS 6.3 ± 6.8). Similar to previous studies, we have demonstrated that patients with COVID-19 have worse functional outcome and morbidity, which is consistent with previous literature (Ntaios et al., 2020). Patients with GGO had worse functional outcome and morbidity in all measures on univariate analysis but when all stroke imaging and clinical features and other co-variates were included in multivariate analysis, GGO was no longer an independent predictor.

### 4.4 Strengths and weaknesses

This was a prospective, pragmatic, multicenter, national study with a large number of institutions and patients recruited from across the country, providing geographic validity. We have also demonstrated that apical GGO is a biomarker with temporal validity at a time point after the first wave of COVID when a mutated variant was predominant (Siddiqui et al., 2020). This study is presented on a background of almost all diagnostic and prognostic COVID-19 models being unreliable without demonstration of a robust performance over time and in varying settings (Wynants et al., 2020). For example, no imaging-based diagnostic and prognostic COVID-19 model developed using machine learning are of potential clinic use (Roberts et al., 2021). In contrast, we provide high level evidence (level 2) that GGO is a reliable and accurate biomarker (Group OLoEW, 2022), and use a simple approach that is robust to both junior and senior assessment.

A limitation of this study is that the virus is continuously mutating, and the dominant strain of the virus is constantly changing. The latest SARS-CoV-2 variant to dominate in the pandemic is Omicron (Whitaker et al., 2022). As with all COVID-19 biomarkers, the performance accuracy shown in the current study is likely to vary over time.

Another limitation is that RT-PCR was used as the reference standard, however it is has limited sensitivity (Jarrom et al., 2022) and therefore the effectiveness of GGO as a biomarker may be underpredicted in this study.

### 4.5 Unanswered questions and future directions

In this prospective study, we have validated that apical GGO is a reliable and accurate diagnostic biomarker in COVID-19. The key finding is that, during a period of high disease prevalence, ground-glass opacification in the lung apices is a reliable diagnostic COVID-19 biomarker with high negative predictive value (97.8 %), especially in vaccinated patients (99.7 %). Ground-glass opacification in the lung apices is associated with worse functional outcome on univariate analysis at discharge but has limited application as a prognostic biomarker in COVID-19 when factors including stroke severity are taken into account.

These findings will be useful in the assessment of suspected stroke patients at the point of COVID-19 triage in ED. However, because the virus is continuously mutating and prevalence is changing, as with all COVID-19 biomarkers, future calibration or validation studies are recommended (Wynants et al., 2020).

## Funding

This study has received funding by the Radiological Research Trust, a non-commercial partner of the National Institute of Health Research (NIHR). TCB was funded by the 10.13039/100010269Wellcome Trust [WT203148/Z/16/Z].

## 6 Compliance with ethical standards

### 6.1 Guarantor

The scientific guarantor of this publication is Dr Thomas Booth.

## 7 Statistics and biometry

No complex statistical methods were necessary for this paper.

## Informed Consent

Written informed consent was waived by the Institutional Review Board.

## Ethical approval

Institutional Review Board approval was obtained.

## 10 Study subjects or cohorts overlap

No study subjects or cohorts have been previously reported.

## 11 Methodology

Methodology: prospective diagnostic or prognostic study multicenter study.

## CRediT authorship contribution statement

**T. Ratneswaren:** Visualization, Conceptualization, Methodology, Funding acquisition, Project adminsitration, Resources, Software, Supervision, Data curation, Validation, Formal analysis, Investigation, Writing – original draft, Writing – review and editing. **N. Chan:** Project administration, Funding acquisition, Data curation, Conceptualization. **J. Aeron-Thomas:** Project administration. **S. Sait:** Data curation. **O. Adesalu:** Data curation. **M. Alhawamdeh:** Data curation. **M. Benger:** Data curation. **J. Garnham:** Data curation. **L. Dixon:** Data curation. **F. Tona:** Data curation. **C. McNamara:** Data curation. **E. Taylor:** Data curation. **K. Lobotesis:** Data curation. **E. Lim:** Data curation. **O. Goldberg:** Data curation. **N. Asmar:** Data curation. **O. Evbuomwan:** Data curation. **S. Banerjee:** Data curation. **L. Holm-Mercer:** Data curation. **J. Senor:** Data curation. **Y. Tsitsiou:** Data curation. **P. Tantrige:** Data curation. **A. Taha:** Data curation. **K. Ballal:** Data curation. **A. Mattar:** Data curation. **A. Daadipour:** Data curation. **K. Elfergani:** Data curation. **R. Barker:** Data curation. **R. Chakravartty:** Data curation. **A.G. Murchison:** Data curation. **B.J. Kemp:** Data curation. **R. Simister:** Conceptualization and Data curation. **I. Davagnanam:** Data curation. **O.Y. Wong:** Data curation. **D. Werring:** Conceptualization and Data curation. **A. Banaras:** Data curation. **M. Anjari:** Data curation. **J.C.L. Rodrigues:** Data curation. **C.A.S. Thompson:** Data curation. **I.R. Haines:** Data curation. **T.A. Burnett:** Data curation. **R.E.Y. Zaher:** Data curation. **V.L. Reay:** Data curation. **M. Banerjee:** Data curation. **C.S.L. Sew Hee:** Data curation. **A.P. Oo:** . **A. Lo:** Data curation. **P. Rogers:** Data curation. **T. Hughes:** Data curation. **A. Marin:** Data curation. **S. Mukherjee:** Data curation. **H. Jaber:** Data curation. **E. Sanders:** Data curation. **S. Owen:** Data curation. **M. Bhandari:** Data curation. **S. Sundayi:** Data curation. **A. Bhagat:** Data curation. **M. Elsakka:** Data curation. **O.H. Hashmi:** Data curation. **M. Lymbouris:** Data curation. **Y. Gurung-Koney:** Data curation. **M. Arshad:** Data curation. **I. Hasan:** Data curation. **N. Singh:** Data curation. **V. Patel:** Data curation. **M. Rahiminejad:** Data curation. **T.C. Booth:** Writing – review & editing, Writing – original draft, Visualization, Validation, Supervision, Software, Resources, Project administration, Methodology, Investigation, Funding acquisition, Formal analysis, Data curation, Conceptualization.

## Declaration of Competing Interest

The authors declare that they have no known competing financial interests or personal relationships that could have appeared to influence the work reported in this paper.

## Data Availability

The data that has been used is confidential.
