# Supplementary material for: COVID-19 Stroke Apical Lung Examination Study 2: a national prospective CTA biomarker study of the lung apices, in patients presenting with suspected acute stroke (COVID SALES 2)
Source: Neuroimage Clin. 2024 Mar 15;42:103590. doi: 10.1016/j.nicl.2024.103590 (PMC10966308; doi:10.1016/j.nicl.2024.103590)
Supplement: Supplementary data 1 [file mmc1.docx]

**Tables**

**Supplementary Table 1- Patient characteristics stratified by RT-PCR result**

|  | **All Patients**  **(n = 1111)** | **RT-PCR negative**  **(n = 883)** | **RT-PCR positive**  **(n = 47)** | ***P* value** |
| --- | --- | --- | --- | --- |
| **Demographics** |  |  |  |  |
| Age (mean, years) | 67.9 ± 16.2 (1111/1111) | 68.9 ± 15.5  (883/883) | 66.8 ± 17.7  (47/47) | 0.38 |
| Sex (female) | 48.2  (535/1111) | 49.0  (883/883) | 46.8  (47/47) | 0.77 |
| Ethnicity |  |  |  |  |
| White | 68.9  (710/1031) | 69.4 (571/823) | 68.9  (31/45) | 0.95 |
| Mix | 1.4  (14/1031) | 1.3  (11/823) | 0.0  (0/45) | 0.44 |
| Asian | 4.9  (51/1031) | 5.0  (41/823) | 8.9  (4/45) | 0.22 |
| Black | 6.6  (68/1031) | 7.0  (57/823) | 4.4  (2/45) | 0.56 |
| Unknown | 15.9  (164/1031) | 14.6 (120/823) | 15.6  (7/45) | 0.57 |
| **Baseline functional status** |  |  |  |  |
| modified Rankin Scale (mRS) (mean) | 0.7 ± 1.2  (1082/1111) | 0.7 ± 1.2  (860/883) | 0.9 ± 1.4  (46/47) | 0.45 |
| **Imaging findings on CT and CTA** |  |  |  |  |
| GGO | 8.5  (94/1111) | 6.3  (56/883) | 59.6  (28/47) | *0.00* |
| Carotid occlusion | 6.7  (74/1111) | 6.2  (55/883) | 14.89  (7/47) | *0.02* |
| Large vessel occlusion | 13.0  (144/1111) | 12.8 (113/883) | 19.1  (9/47) | 0.21 |
| Medium vessel occlusion | 9.8  (109/1111) | 10.4  (92/883) | 10.6  (5/47) | 0.96 |
| Tandem occlusion | 2.8  (30/1111) | 2.49  (22/883) | 4.3  (2/47) | 0.46 |
| Clot length (mm) | 27.4 ± 65.8 (211/1111) | 28.02 ± 70.4  (185/883) | 23.0 ± 39.4  (14/47) | 0.79 |
| Acute infarct | 23.4  (260/1111) | 23.8 (210/883) | 40.4  (19/47) | *0.01* |
| ASPECTS score (mean) | 9.4 ± 1.5  (1111/1111) | 9.4 ± 1.5  (883/883) | 8.6 ± 2.4  (47/47) | *0.00* |
| **Symptoms** |  |  |  |  |
| Symptomatic fever | 3.4  (34/1008) | 2.3  (18/800) | 33.3  (14/42) | *0.00* |
| Cough | 5.4  (52/984) | 4.1  (32/782) | 34.2  (14/40) | *0.00* |
| Fatigue | 6.9  (61/887) | 5.96  (42/705) | 29.3  (12/41) | *0.00* |
| Shortness of breath | 4.4  (40/915) | 3.0  (22/729) | 26.8  (11/41) | *0.00* |
| Myalgia | 4.5  (40/884) | 3.9  (27/702) | 25.0  (10/40) | *0.00* |
| **Signs** |  |  |  |  |
| GCS (mean) | 14.2 ± 1.9  (1037/1111) | 14.2 ± 1.9  ( 831/883) | 13.3 ± 2.9  (47/47) | *0.00* |
| NIHSS (mean) | 6.6 ± 7.0  (989/1111) | 6.9 ± 6.9  (791/883) | 9.7 ± 9.0  (42/47) | *0.01* |
| Heart rate (beats per minute) | 81.8 ± 16.8 (1059/1111) | 81.6 ± 16.6  (853/883) | 85.6 ± 15.3  (47/47) | 0.10 |
| Respiratory rate (breaths per minute) | 18.4 ± 4.0 (1056/1111) | 18.45 ± 4.2  (853/883) | 18.78 ± 3.0  (46/47) | 0.60 |
| Systolic blood pressure (mmHg) | 150.8 ± 28.5 (1063/1111) | 151.39 ± 28.0  (855/883) | 146.6 ± 27.4  (47/47) | 0.26 |
| Diastolic blood pressure (mmHg) | 84.8 ± 17.8 (1061/1111) | 84.8 ± 17.4  (853/883) | 81.5 ± 15.6  (47/47) | 0.21 |
| Core body temperature (mean, ℃) | 36.7 ± 1.2 (1054/1111) | 36.6 ± 1.3  (850/883) | 37.2 ± 1.1  (47/47) | *0.00* |
| Oxygen saturations (mean, %) | 97.1 ± 2.2 (1059/1111) | 97.2 ± 2.1  (853/883) | 95.3 ± 3.0  (47/47) | *0.00* |
| Supplementary oxygen required (%) | 4.3  (48/1111) | 4.2  (37/883) | 6.4  (3/47) | 0.44 |
| **Comorbidities** |  |  |  |  |
| Hypertension | 51.3  (564/1111) | 52.1  (456/875) | 48.9  (23/47) | 0.67 |
| Diabetes | 21.9  (241/1099) | 22.6 (198/875) | 27.7  (13/47) | 0.42 |
| Cardiovascular disease | 21.6  (237/1099) | 21.3 (186/875) | 21.3  (10/47) | 1.00 |
| Atrial fibrillation | 17.1  (188/1098) | 18.3 (160/874) | 8.5  (4/47) | 0.09 |
| Hypercholesterolemia | 25.9  (282/1087) | 27.9 (241/865) | 19.2  (9/47) | 0.19 |
| Sickle cell disease | 0.8  (8/1071) | 0.94  (8/850) | 0.0  (0/47) | 0.50 |
| Body Mass Index | 27.3 ± 9.9 (592/1111) | 27.7 ± 10.6  (485/883) | 29.0 ± 7.3  (29/47) | 0.16 |
| Smoking status | 32.2  (326/1013) | 33.1  (8/850) | 26.7  (12/45) | 0.37 |
| **Past medical history of stroke** | 27.3  (286/1082) | 29.0 (241/861) | 17.0  (8/47) | 0.10 |
| **Family history of stroke** | 5.4  (54/995) | 5.4  (42/782) | 2.2  (1/46) | 0.34 |
| **At least one vaccine dose** | 58.5  (419/716) | 59.2 (346/584) | 28.0  (7/25) | *0.00* |
| **Treatment** |  |  |  |  |
| Thrombolysis | 18.6 (207/1111) | 20.4 (180/883) | 17.0  (8/47) | 0.58 |
| Time from door to thrombolysis (minutes, mean) | 64.6 ± 49.6 (190/1111) | 63.1 ± 50.2  (164/883) | 88 ± 23.6  (7/47) | 0.19 |
| Thrombectomy | 5.6  (62/1111) | 5.7  (50/883) | 2.1  (1/47) | 0.30 |

Data are mean ± standard deviation or % (n/n).

Data in *italics* are statistically significant at the p < 0.05 significance level.

RT-PCR = Reverse Transcriptase Polymerase Chain Reaction swab test. mRS = modified Rankin Scale. CT = Computed Tomography. CTA = CT Angiography. GGO = Ground-Glass Opacification. ASPECTS = Alberta Stroke Program Early CT score. GCS = Glasgow Coma Scale. NIHSS = National Institute of Health Stroke Score.

Carotid occlusion is defined as occlusion of the common carotid artery or the cervical, petrous or cavernous segments of the internal carotid artery.

Large vessel occlusion is defined as intra-cranial occlusion of a major intracranial vessel including the distal internal carotid artery (ophthalmic, posterior communicating, anterior choroidal and terminal segments), the middle cerebral artery at M1 (prior to the bifurcation), anterior cerebral artery at A1, vertebral artery at V4, basilar artery or posterior cerebral artery at P1.

Medium vessel occlusion is defined as vascular occlusion of the middle cerebral artery (M2/3), anterior cerebral artery (A2/3) or posterior cerebral artery (P2/3).

**Supplementary Table 2- Analysis of ground-glass opacification as a diagnostic biomarker in patients with a RT-PCR result**

|  | **PPV %**  **(95% CI)** | **NPV %**  **(95% CI)** | **Sensitivity % (95% CI)** | **Specificity % (95% CI)** |
| --- | --- | --- | --- | --- |
| **Population as a whole (930/1111)** | 33.3  (23.4 - 44.5) | 97.8  (96.5 - 98.6) | 59.6  (44.3 - 73.6) | 93.7  (91.8 - 95.2) |
| **Unvaccinated subgroup**  **(256/609)** | 46.2  (26.6 – 66.6) | 97.4  (94.4-99.0) | 66.7  (41.0 – 86.7) | 94.1  (90.3 – 96.7) |
| **Vaccinated subgroup**  **(353/609)** | 20.0  (7.7 - 38.6) | 99.7  (98.3 - 100) | 85.7  (42.1 - 99.6) | 93.1  (89.8 - 95.5) |
| **Junior Radiologist (687/1860)** | 30.3  (19.6 - 42.9) | 97.1  (95.5 - 98.3) | 52.6  (35.8 - 69.0) | 92.9  (90.7 - 94.8) |
| **Senior Radiologist (1173/1860)** | 30.8  (22.3 - 40.5) | 97.8  (96.8 - 98.6) | 58.9  (45.0 - 71.9) | 93.4  (92.8 - 94.8) |

Data are % with 95% confidence interval.

PPV = positive predictive value. NPV = negative predictive value.

Junior radiologists are radiology trainees (UK registrar grade; US resident equivalent) who have not obtained the UK’s radiology licensure examination (Fellowship of the Royal College of Radiologists which is similar to board certification in the US). Senior radiologists are those who have passed the UK’s radiology licensure examination.

**Supplementary Table 3- Adjusted odds ratio for obtaining a positive RT-PCR result**

|  | **OR (95% CI)** | ***P* value** |
| --- | --- | --- |
| **Carotid occlusion** | 1 (0.2 - 5.3) | 1.00 |
| **Acute infarct** | 2.3 (0.6 - 8.2) | 0.20 |
| **ASPECTS** | 1.3 (0.9 - 1.9) | 0.19 |
| **NIHSS** | 1.0 (1.0 - 1.1) | 0.40 |
| **GCS** | 1.0 (0.7 - 1.4) | 0.88 |
| **Symptomatic fever** | 1.7 (0.4 - 7.3) | 0.49 |
| **Cough** | 2.4 (0.7 - 8.9) | 0.19 |
| **Fatigue** | 0.5 (0.1 - 2.6) | 0.38 |
| **Shortness of breath** | 1.9 (0.3 - 12.6) | 0.49 |
| **Myalgia** | 8.9 (2.1 - 38.2) | *0.00* |
| **Core body temperature** | 1.9 (1.1 - 3.2) | *0.01* |
| **Oxygen saturations** | 0.9 (0.7 - 1.1) | 0.13 |
| **GGO** | 15.7 (6.2 - 40.1) | *0.00* |

Data are presented as odds ratios with 95% confidence intervals.

Data in *italics* are statistically significant at the p < 0.05 significance level.

ASPECTS = Alberta Stroke Program Early CT score. NIHSS = National Institute of Health Stroke Score. GCS = Glasgow Coma Scale. GGO = Ground-Glass Opacification.

**Supplementary Table 4- Patient characteristics stratified by ground-glass opacification**

|  | **GGO absent**  **(n = 1017)** | **GGO present**  **(n = 94)** | ***P* value** |
| --- | --- | --- | --- |
| **Demographics** |  |  |  |
| Age (mean, years) | 67.7 ± 16.3 (1017/1017) | 70.1 ± 15.6 (94/94) | 0.16 |
| Sex (female) | 47.6  (484/1017) | 54.3  (51/94) | 0.22 |
| Ethnicity |  |  |  |
| White | 69.2  (650/939) | 65.2  (60/92) | 0.43 |
| Mix | 1.3  (12/939) | 2.2  (2/92) | 0.46 |
| Asian | 5.0  (47/939) | 4.3  (4/92) | 0.84 |
| Black | 6.6  (62/939) | 6.5  (6/92) | 0.98 |
| Unknown | 16.1  (152/939) | 13.0  (12/92) | 0.95 |
| **Baseline functional status** |  |  |  |
| modified Rankin Scale (mRS) (mean) | 0.7 ± 1.2 (989/1017) | 1.0 ± 1.4  (93/94) | *0.04* |
| **Imaging findings on CT and CTA** |  |  |  |
| Carotid occlusion | 6.0  (61/1017) | 13.8  (13/94) | *0.00* |
| Large vessel occlusion | 12.6  (128/1017) | 17.0  (16/94) | 0.22 |
| Medium vessel occlusion | 9.1  (93/1017) | 17.0  (16/94) | *0.01* |
| Tandem occlusion | 2.5  (25/1017) | 5.3  (5/94) | 0.10 |
| Clot length (mm) | 27.7 ± 69.2 (192/1017) | 25.4 ± 36.1 (29/94) | 0.86 |
| Acute infarct | 22.6  (230/1017) | 31.9  (30/94) | *0.04* |
| ASPECTS score (mean) | 9.5 ± 1.4 (1017/1017) | 8.8 ± 2.4  (94/94) | *0.00* |
| **Symptoms** |  |  |  |
| Symptomatic fever | 2.1  (19/921) | 17.2  (15/87) | *0.00* |
| Cough | 3.8  (34/899) | 21.2  (18/85) | *0.00* |
| Fatigue | 5.4  (44/810) | 22.1  (17/77) | *0.00* |
| Shortness of breath | 3.0  (25/837) | 19.2  (15/78) | *0.00* |
| Myalgia | 3.3  (27/809) | 17.3  (13/75) | *0.00* |
| **Signs** |  |  |  |
| GCS (mean) | 14.2 ± 1.9 (952/1017) | 13.8 ± 2.3 (84/94) | *0.03* |
| NIHSS (mean) | 6.3 ± 6.8 (910/1017) | 9.0 ± 8.6  (79/94) | *0.00* |
| Heart rate (beats per minute) | 81.3 ± 16.9 (972/1017) | 87.1 ± 16.0 (87/94) | *0.00* |
| Respiratory rate (breaths per minute) | 18.3 ± 4.1 (968/1017) | 18.6 ± 2.9 (88/94) | 0.61 |
| Systolic blood pressure (mmHg) | 151.3 ± 28.5 (976/1017) | 145.4 ± 28.4 (87/94) | 0.06 |
| Diastolic blood pressure (mmHg) | 85.1 ± 17.9 (974/1017) | 81.6 ± 15.8 (87/94) | 0.08 |
| Core body temperature (mean, ℃) | 36.6 ± 1.2 (967/1017) | 37.0 ± 0.95 (87/94) | *0.00* |
| Oxygen saturations (mean, %) | 97.2 ± 2.1 (971/1017) | 96.1 ± 2.6 (88/94) | *0.00* |
| Supplementary oxygen required (%) | 3.6  (1017/1017) | 11.7  (94/94) | *0.00* |
| **Body mass index** | 27.4 ± 10.2 (539/1017) | 29.0 ± 7.2 (53/94) | *0.03* |
| **Comorbidities** |  |  |  |
| Hypertension | 50.8  (511/1006) | 57.0  (53/93) | 0.25 |
| Diabetes | 21.5  (216/1006) | 26.9  (25/93) | 0.23 |
| Cardiovascular disease | 21.0  (211/1006) | 28.0  (26/93) | 0.12 |
| Atrial fibrillation | 16.7  (168/1005) | 21.5  (20/93) | 0.24 |
| Hypercholesterolemia | 25.7  (256/997) | 28.9  (26/90) | 0.24 |
| Sickle cell disease | 0.9  (9/980) | 0.0  (0/91) | 0.36 |
| Body Mass Index | 27.4 ± 10.2 (539/1017) | 29.0 ± 7.2 (53/94) | *0.03* |
| Smoking status | 32.0  (298/931) | 34.2  (28/82) | 0.69 |
| **Past medical history of stroke** | 26.8  (266/993) | 23.6  (21/89) | 0.49 |
| **Family history of stroke** | 5.5  (50/913) | 4.9  (4/82) | 0.82 |
| **At least one vaccine dose** | 58.9  (387/657) | 54.2  (32/59) | 0.49 |
| **RT-PCR positive** | 2.2  (19/846) | 33.3  (28/84) | *0.00* |
| **Treatment** |  |  |  |
| Thrombolysis | 18.7  (190/1017) | 18.1  (17/94) | 0.88 |
| Time from door to thrombolysis (minutes, mean) | 64.0 ± 50.4 (176/190) | 71.8 ± 38.5 (14/17) | 0.57 |
| Thrombectomy | 5.7  (58/1017) | 4.3  (4/94) | 0.56 |

Data are mean ± standard deviation or % (n/n).

Data in *italics* are statistically significant at the p < 0.05 significance level.

Five results in *red italics* are additional group differences compared to stratification by RT-PCR in Table 1. The result in blue is not significant, demonstrating no significant group difference compared to stratification by RT-PCR in Table 1.

RT-PCR = Reverse Transcriptase Polymerase Chain Reaction swab test. mRS = modified Rankin Scale. CT = Computed Tomography. CTA = CT Angiography. GGO = Ground-Glass Opacification. ASPECTS = Alberta Stroke Program Early CT score. GCS = Glasgow Coma Scale. NIHSS = National Institute of Health Stroke Score.

**Supplementary Table 5- Outcome data including symptomatic hemorrhage, length of stay and functional outcome, stratified by RT-PCR result and GGO presence.**

| **Symptomatic hemorrhage** | | |
| --- | --- | --- |
| **RT-PCR negative** | **RT-PCR positive** | *P* value |
| 3.8 ± 19.1  (33/869) | 2.2 ± 14.9  (1/45) | 0.59 |
| **GGO absent** | **GGO present** | *P* value |
| 4.0 ± 19.6  (40/997) | 1.1 ± 10.4  (1/92) | 0.16 |
| **Length of stay in days** | | |
| **RT-PCR negative** | **RT-PCR positive** | *P* value |
| 12.7 ± 29.6  (759/869) | 12.2 ± 17.4  (37/45) | 0.92 |
| **GGO absent** | **GGO present** | *P* value |
| 12.1 ± 29.2  (869/997) | 17.9 ± 36.1  (72/92) | 0.11 |
| **Unadjusted Odds Ratio of Independent mRS (0-2) at discharge** | | |
| **RT-PCR positive** |  | *P* value |
| 0.54  (0.29 - 0.99) |  | *0.04* |
| **GGO present** |  | *P* value |
| 0.48  (0.31 - 0.74) |  | *0.00* |
| **Adjusted Odds Ratio of Independent mRS (0-2) at discharge** | | |
| **RT-PCR positive** |  | *P* value |
| 1.02  (0.25 - 4.26) |  | 0.97 |
| **GGO present** |  | *P* value |
| 0.46  (0.19 - 1.15) |  | 0.10 |
| **Unadjusted Odds Ratio of mRS shift at discharge** | | |
| **RT-PCR positive** |  | *P* value |
| 1.16  (0.64 - 2.07) |  | 0.62 |
| **GGO present** |  | *P* value |
| 1.79  (1.19 - 2.68) |  | *0.00* |
| **Adjusted Odds Ratio of mRS shift at discharge** | | |
| **RT-PCR positive** |  | *P* value |
| 6.32  (1.30- 30.9) |  | 0.02 |
| **GGO present** |  | *P* value |
| 1.57  (0.60-4.14) |  | 0.36 |

Data are % ± standard deviation (n/n) or odds ratio (95% confidence interval).

Data in *italics* are statistically significant at the p < 0.05 significance level.

RT-PCR = Reverse Transcriptase Polymerase Chain Reaction swab test. mRS = modified Rankin Scale.. GGO = Ground-Glass Opacification.

Odds ratio of independent mRS at discharge is adjusted for age, black ethnicity, baseline mRS, carotid occlusion, large vessel occlusion, medium vessel occlusion, infarct, ASPECTS, GCS, NIHSS, symptomatic fever, respiratory rate, systolic blood pressure, diastolic blood pressure, supplementary oxygen, oxygen saturations and body mass index.

Odds ratio of mRS shift at discharge is adjusted for age, black ethnicity, baseline mRS, carotid occlusion, large vessel occlusion, medium vessel occlusion, infarct, ASPECTS, GCS, NIHSS, systolic blood pressure, diastolic blood pressure, supplementary oxygen, oxygen saturations and body mass index.

**Supplementary Table 6- Univariate survival analysis at 30 days**

| **Univariate analysis** | **᙭^2^ or Hazard Ratio** | ***P* value** |
| --- | --- | --- |
| Age | 1.03 | *0.00* |
| Sex (female) | 6.70 | *0.01* |
| Ethnicity |  |  |
| White | 1.09 | 0.30 |
| Mix | 0.23 | 0.63 |
| Asian | 0.41 | 0.52 |
| Black | 2.80 | 0.10 |
| Unknown | 1.31 | 0.52 |
| modified Rankin Scale (mRS) | 1.15 | 0.17 |
| **Imaging findings on CT and CTA** |  |  |
| Carotid occlusion | 16.88 | *0.00* |
| Large vessel occlusion | 7.99 | *0.00* |
| Medium vessel occlusion | 3.62 | 0.06 |
| Tandem occlusion | 15.06 | *0.00* |
| Clot length | 1.01 | 0.87 |
| Infarct | 6.64 | *0.01* |
| ASPECTS | 0.81 | *0.00* |
| GGO | 5.65 | *0.02* |
| **Symptoms** |  |  |
| Symptomatic fever | 1.99 | 0.16 |
| Cough | 7.24 | *0.01* |
| Fatigue | 0.32 | 0.57 |
| Shortness of breath | 10.81 | *0.00* |
| Myalgia | 0.81 | 0.39 |
| **Signs** |  |  |
| GCS | 0.75 | *0.00* |
| NIHSS | 1.07 | *0.00* |
| Heart rate | 1.01 | 0.10 |
| Respiratory rate | 1.05 | *0.03* |
| Systolic blood pressure | 1.00 | 0.48 |
| Diastolic blood pressure | 1.01 | 0.15 |
| Core body temperature | 0.93 | 0.44 |
| Oxygen saturations | 0.88 | *0.00* |
| Supplementary oxygen required (%) | 37.6 | *0.00* |
| **Comorbidities** |  |  |
| Hypertension | 0.05 | 0.82 |
| Diabetes | 0.66 | 0.42 |
| Cardiovascular disease | 0.48 | 0.49 |
| Atrial fibrillation | 0.99 | 0.32 |
| Hypercholesterolaemia | 0.04 | 0.83 |
| Sickle cell disease | 0.71 | 0.40 |
| Smoking status | 0.00 | 0.95 |
| **Past medical history of stroke** | 0.33 | 0.84 |
| **Family history of stroke** | 1.17 | 0.28 |
| **Body Mass Index** | 0.96 | 0.23 |
| **First vaccine dose** | 0.88 | 0.35 |
| **RT-PCR positive** | 0.22 | 0.64 |
| **Treatment** |  |  |
| Thrombolysis | 0.59 | 0.44 |
| Thrombectomy | 2.58 | 0.11 |

Data in *italics* are statistically significant at the p < 0.05 significance level.

mRS = modified Rankin Scale. CT = Computed Tomography. CTA = CT Angiography. GGO = Ground-Glass Opacification. ASPECTS = Alberta Stroke Program Early CT score. GCS = Glasgow Coma Scale. NIHSS = National Institute of Health Stroke Score. RT-PCR = Reverse Transcriptase Polymerase Chain Reaction swab test.

**Supplementary Table 7- Univariate survival analysis at 90 days**

| **Univariate analysis** | **᙭^2^ or Hazard Ratio** | ***P* value** |
| --- | --- | --- |
| Mean Age | 1.03 | *0.00* |
| Sex (female) | 9.53 | *0.00* |
| Ethnicity |  |  |
| White | 2.18 | 0.14 |
| Mix | 0.21 | 0.64 |
| Asian | 0.57 | 0.45 |
| Black | 3.56 | 0.06 |
| Unknown | 2.65 | 0.10 |
| **Baseline functional status** | 1.14 | 0.15 |
| **Imaging findings on CT and CTA** |  |  |
| Carotid occlusion | 15.34 | *0.00* |
| Large vessel occlusion | 6.42 | *0.01* |
| Medium vessel occlusion | 2.77 | 0.10 |
| Tandem occlusion | 16.17 | *0.00* |
| Clot length | 1.00 | 0.37 |
| Infarct | 5.02 | *0.03* |
| ASPECTS | 0.83 | *0.00* |
| GGO | 4.02 | *0.04* |
| **Symptoms** |  |  |
| Symptomatic fever | 3.16 | 0.08 |
| Cough | 6.21 | *0.01* |
| Fatigue | 0.23 | 0.63 |
| Shortness of breath | 13.56 | *0.00* |
| Myalgia | 0.39 | 0.53 |
| **Signs** |  |  |
| GCS | 0.84 | *0.00* |
| NIHSS | 1.07 | *0.00* |
| Heart rate | 1.01 | 0.09 |
| Respiratory rate | 1.05 | 1.00 |
| Systolic blood pressure | 1.00 | 0.81 |
| Diastolic blood pressure | 1.01 | 0.35 |
| Core body temperature | 0.92 | 0.32 |
| Supplementary oxygen required (%) | 29.9 | *0.00* |
| Oxygen saturations | 0.89 | *0.00* |
| **Comorbidities** |  |  |
| Hypertension | 0.00 | 0.97 |
| Diabetes | 0.88 | 0.35 |
| Cardiovascular disease | 0.31 | 0.58 |
| Atrial fibrillation | 2.53 | 0.11 |
| Hypercholesterolaemia | 0.01 | 0.93 |
| Sickle cell disease | 0.81 | 0.37 |
| Smoking status | 0.00 | 0.99 |
| **Past medical history of stroke** | 0.19 | 0.91 |
| **Family history of stroke** | 1.30 | 0.26 |
| **Body Mass Index** | 0.95 | 0.13 |
| **RT-PCR positive** | 0.29 | 0.59 |
| **First vaccine dose** | 0.45 | 0.50 |
| **Treatment** |  |  |
| Thrombolysis | 1.70 | 0.19 |
| Thrombectomy | 1.68 | 0.19 |

Data in *italics* are statistically significant at the p < 0.05 significance level.

mRS = modified Rankin Scale. CT = Computed Tomography. CTA = CT Angiography. GGO = Ground-Glass Opacification. ASPECTS = Alberta Stroke Program Early CT score. GCS = Glasgow Coma Scale. NIHSS = National Institute of Health Stroke Score. RT-PCR = Reverse Transcriptase Polymerase Chain Reaction swab test.

**Supplementary Table 8- Multivariate survival analysis at 30 days**

|  | **Hazard Ratio (95% CI)** | ***P* value** |
| --- | --- | --- |
| Age | 1.01 (0.99 - 1.04) | 0.33 |
| Sex (female) | 1.46 (0.70 - 3.00) | 0.31 |
| Carotid occlusion | 2.19 (0.77 - 6.21) | 0.14 |
| Large vessel occlusion | 0.78 (0.30 - 2.05) | 0.62 |
| Tandem occlusion | 0.86 (0.17 - 4.43) | 0.86 |
| Infarct | 0.56 (0.18 - 1.75) | 0.32 |
| ASPECTS | 0.87 (0.69 - 1.09) | 0.23 |
| GGO | 1.37 (0.51 - 3.67) | 0.53 |
| GCS | 0.94 (0.78 - 1.12) | 0.47 |
| NIHSS | 1.03 (0.97 - 1.09) | 0.40 |
| Cough | 0.66 (0.20 - 2.12) | 0.48 |
| Shortness of breath | 1.65 (0.45 - 6.09) | 0.45 |
| Respiratory rate | 0.99 (0.88 - 1.11) | 0.83 |
| Supplementary oxygen required (%) | 5.07 (2.03 - 12.65) | *0.00* |
| Oxygen saturations | 0.9 (0.83 - 0.98) | *0.01* |

Data in *italics* are statistically significant at the p < 0.05 significance level.

ASPECTS = Alberta Stroke Program Early CT score. GGO = Ground-Glass Opacification. GCS = Glasgow Coma Scale. NIHSS = National Institute of Health Stroke Score.

Multivariate survival analysis at 30 days, adjusted for age, gender, carotid occlusion, large vessel occlusion, tandem, infarct, aspects, GGO, GCS, NIHSS, cough, shortness of breath, respiratory rate, oxygen saturations and supplemental oxygen.

**Supplementary Table 9- Multivariate survival analysis at 90 days**

|  | **Hazard Ratio (95% CI)** | ***P* value** |
| --- | --- | --- |
| Age | 1.02 (0.99 - 1.04) | 0.18 |
| Sex (female) | 1.63 (0.81 - 3.28) | 0.17 |
| Carotid occlusion | 2.15 (0.79 - 5.88) | 0.14 |
| Large vessel occlusion | 0.6 (0.23 - 1.58) | 0.30 |
| Tandem occlusion | 0.87 (0.17 - 4.46) | 0.86 |
| Infarct | 0.53 (0.17 - 1.61) | 0.26 |
| ASPECTS | 0.86 (0.69 - 1.08) | 0.20 |
| GGO | 1.21 (0.45 - 3.23) | 0.71 |
| GCS | 0.92 (0.79 - 1.08) | 0.30 |
| NIHSS | 1.02 (0.97 - 1.08) | 0.44 |
| Cough | 0.7 (0.22 - 2.20) | 0.54 |
| Shortness of breath | 1.85 (0.53 - 6.46) | 0.33 |
| Supplementary oxygen required (%) | 4.33 (1.81 - 10.39) | *0.00* |
| Oxygen saturations | 0.92 (0.85 - 0.99) | *0.02* |

Data in *italics* are statistically significant at the p < 0.05 significance level.

ASPECTS = Alberta Stroke Program Early CT score. GGO = Ground-Glass Opacification. GCS = Glasgow Coma Scale. NIHSS = National Institute of Health Stroke Score.

Multivariate survival analysis at 90 days adjusted for age, gender, carotid occlusion, large vessel occlusion, tandem, infarct, aspects, GGO, GCS, NIHSS, cough, shortness of breath, supplemental oxygen and oxygen saturations.
